# Cysteine and hydrogen sulphide in the regulation of metabolism: insights from genetics and pharmacology

**DOI:** 10.1002/path.4659

**Published:** 2015-11-13

**Authors:** Roderick N Carter, Nicholas M Morton

**Affiliations:** ^1^ Molecular Metabolism Group, University/BHF Centre for Cardiovascular Sciences, Queens Medical Research InstituteUniversity of EdinburghUK

**Keywords:** cysteine, sulphide, obesity, diabetes, genetic models, metabolism, insulin resistance, adipose, liver

## Abstract

Obesity and diabetes represent a significant and escalating worldwide health burden. These conditions are characterized by abnormal nutrient homeostasis. One such perturbation is altered metabolism of the sulphur‐containing amino acid cysteine. Obesity is associated with elevated plasma cysteine, whereas diabetes is associated with reduced cysteine levels. One mechanism by which cysteine may act is through its enzymatic breakdown to produce hydrogen sulphide (H_2_S), a gasotransmitter that regulates glucose and lipid homeostasis. Here we review evidence from both pharmacological studies and transgenic models suggesting that cysteine and hydrogen sulphide play a role in the metabolic dysregulation underpinning obesity and diabetes. We then outline the growing evidence that regulation of hydrogen sulphide levels through its catabolism can impact metabolic health. By integrating hydrogen sulphide production and breakdown pathways, we re‐assess current hypothetical models of cysteine and hydrogen sulphide metabolism, offering new insight into their roles in the pathogenesis of obesity and diabetes. © 2015 The Authors. Pathological Society of Great Britain and Ireland.

## Obesity and diabetes

Obesity and type 2 diabetes are a major global health burdens that have dramatically increased in prevalence in the last few decades. According to the World Health Organization, worldwide obesity has more than doubled since 1980 1. As of 2014 it was estimated that 600 million worldwide could be classed obese, almost 10% of the global population 1. Type 2 diabetes has become similarly common, with a global prevalence of 9% in those over 18 years of age 2. The two conditions are strongly associated 3, largely due to obesity being a strong risk factor for the development of insulin resistance – a key underlying mechanism in type 2 diabetes 4. This relationship is not absolute, and many individuals remain metabolically healthy in spite of having high fat mass, while others may suffer diabetes despite normal fat mass 5. A cardinal feature of both obesity and type 2 diabetes is atypical carbohydrate and lipid metabolism. Nutritional excess eventually overloads the expanding adipose tissue, leading to ectopic accumulation of lipid in key metabolic tissues such as the liver, muscle and insulin‐producing pancreatic islets 6, 7. This produces a lipotoxic stress 8 that leads to inflammation and insulin‐resistance in the adipose tissue, liver and muscle, with a consequent dysregulation of glucose homeostasis that precipitates diabetes. Specifically, adipose tissue free fatty acid release is increased (a process normally suppressed by insulin after feeding) and glucose metabolism is skewed towards lipogenesis 9. In the liver, insulin‐resistance increases hepatic production of glucose by gluconeogenesis and glycogen breakdown (which is normally suppressed by post‐prandial insulin action), contributing to hyperglycaemia. Of note, lipid synthesis and transport pathways remain sensitive to insulin in the liver, driving lipogenesis and altering lipoprotein profiles that ultimately contribute to atherosclerosis 10. Insulin resistance in muscle leads to impaired glucose disposal and oxidation, thus exacerbating hyperglycaemia. Increased demand for insulin engendered by hyperglycaemia eventually leads to pancreatic β cell exhaustion, insulin deficiency and frank diabetes.

## Links between cysteine and obesity and diabetes

An emerging factor associated with obesity is altered levels of the sulphur‐containing amino acid cysteine. Plasma total cysteine correlates positively with obesity, as defined by body mass index 11, 12, and in particular with fat mass 13. This relationship appears to be specific to cysteine, rather than amino acids in general, as no other amino acid shares the same strength of association with obesity 14. Plasma levels of other amino acids that associate with obesity, such as cystathionine and glutamate, normalize following gastric bypass surgery 15; by contrast, plasma cysteine levels remain high, supporting the hypothesis that elevated cysteine is not merely the consequence of increased fat mass but may mechanistically underlie fat gain. This concept is supported by evidence that in some rodent models, increasing dietary cysteine levels can result in increased adiposity 16. The mechanism by which cysteine could cause increased tissue fat accumulation remains unresolved; one possibility is that cysteine directly regulates energy expenditure and/or appetite. Conflicting with its proposed role in fat mass gain, there is evidence that cysteine reduces appetite in humans and in rodent models 17. Alternatively, it has been reported that dietary cystine reduces metabolic rate in mice, consistent with increased energy storage 18. Understanding any mechanism of action of cysteine with respect to wider metabolic control must also take into account that, contrary to its relationship with obesity, type 2 diabetes has, in some studies, been associated with lower plasma cysteine levels 19.

## Hydrogen sulphide as a metabolite of cysteine; clinical correlates with obesity/diabetes

In addition to the potential effects of cysteine *per se*, it is possible that metabolites of cysteine could be crucial. In this regard, cysteine is a critical substrate for the intracellular generation of hydrogen sulphide (H_2_S), an enzymatically produced physiologically active gasotransmitter. Hydrogen sulphide has emerged as an important factor in the modulation of insulin action in tissues such as liver, adipose tissue and islets of Langerhans. In contrast to plasma total cysteine, which has a positive correlation to obesity and adiposity 14, plasma hydrogen sulphide has been found to be negatively correlated with measures of adiposity, in particular waist circumference and waist:hip ratio 20. In this study, hydrogen sulphide was lowest in subjects with obesity and type 2 diabetes, although regression analysis suggested that the adiposity was the main driver for predicting low plasma hydrogen sulphide. Lower plasma hydrogen sulphide levels were confirmed independently in another cohort of type 2 diabetes subjects 21. Thus, cysteine and hydrogen sulphide have opposing relationships in obesity but share the same negative correlation with type 2 diabetes. It should be noted that, as accurate hydrogen sulphide measurements from plasma are technically challenging 22, these findings remain preliminary.

## Hydrogen sulphide: production, breakdown and mechanisms of action

**Figure 1:**
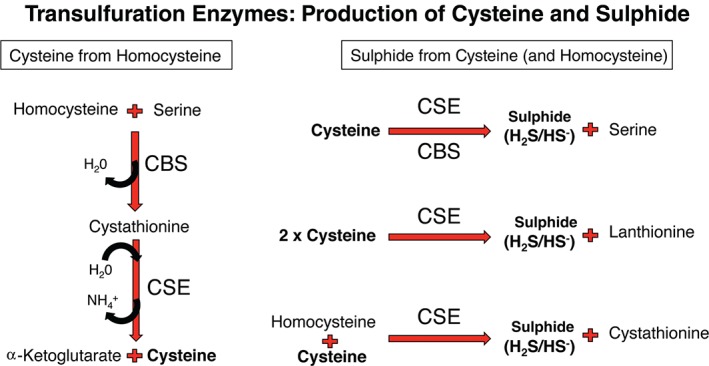
Transulphuration – a source of cysteine and sulphide. CBS and CSE link cysteine generation from homocysteine, an intermediate of the methionine cycle, with sulphide generation. CBS and CSE can be regulated at substrate, protein and RNA levels, and thus dynamic changes in the flux of both cysteine and sulphide production is predicted to be possible in tissues

Hydrogen sulphide was first discovered to be an endogenously produced, physiologically active compound in rodent brain 23, 24, and previously was regarded as a lethal respiratory toxin. A significant route of hydrogen sulphide production is the reverse transulphuration system, whereby homocysteine is converted to cysteine via cystathionine. The two steps of this metabolic conversion are carried out by the predominantly cytosolic enzymes cystathionine β‐synthase (CBS) and cystathionine γ‐lyase (CSE; also referred to as CTH) 25, 26. Both CBS and CSE are capable of a number of related but distinct enzymatic conversions, with hydrogen sulphide formed as a product of some of these reactions 27, 28 (Figure 1). The relative contribution of these two enzymes to hydrogen sulphide production are dependent on tissue‐specific enzyme levels, substrate and co‐factor availability, and activators such as *S*‐adenosyl methionine 27, 28. Mercaptopyruvate sulphur transferase (MPST; also referred to as MST) may also generate hydrogen sulphide through the cysteine transamination product 3‐mercaptopyruvate 29. MPST acquires the sulphur atom from mercaptopyruvate to form a persulphide intermediate, which is released as hydrogen sulphide in the presence of thioredoxin or other reducing agents 30.

**Figure 2:**
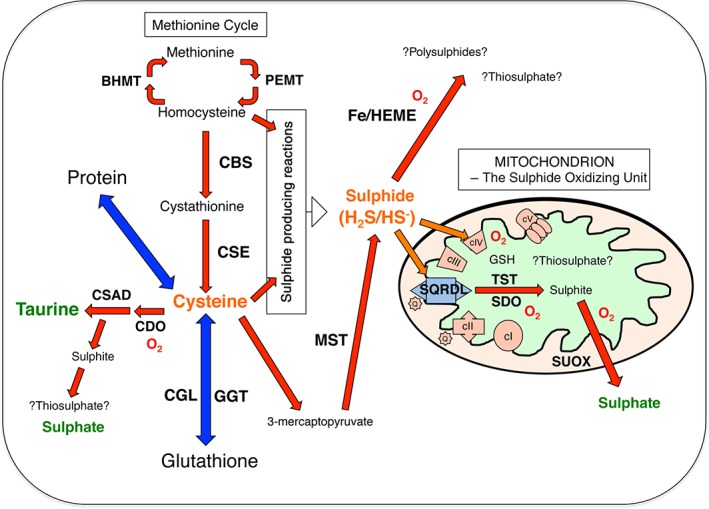
A model for cysteine and sulphide metabolism: bold capitals, enzymes discussed in this review; red arrows, chemical transformations; a red O_2_ is indicated where oxygen is consumed; orange arrows, movement of sulphide to mitochondrial components; blue arrows, equilibrium of cysteine with protein and glutathione pools; green, main urinary excreted end products of sulphur metabolism; thiosulphate and polysulphide metabolism remain controversial and are indicated as such with question marks; not indicated is the fact that methionine and cysteine can enter the system as part of protein‐containing diets. It is hypothesized that the balance of dietary intake, and flux through these pathways, influences the steady state of cysteine and sulphide and thereby contributes to metabolic control of the cell

Significantly, hydrogen sulphide breakdown is often overlooked, but has the potential to regulate physiological levels and may be important in preventing toxic accumulation in tissues. Clearance of hydrogen sulphide can occur by at least two mechanisms; the most characterized is oxidation by mitochondria through a respiratory route (Figure 2). This pathway has established hydrogen sulphide as the first non‐carbon‐based respiratory fuel in mammalian systems 31. The proposed canonical breakdown pathway involves sulphide quinone oxidoreductase (SQR; also referred to as SQRDL), persulphide dioxygenase (also referred to as Ethe1, or sulphite dioxygenase), and thiosulphate sulphur transferase (TST; also referred to as rhodanese), 32, 33. The main products of this oxidation appear to be sulphite and thiosulphate. Sulphite itself can be further oxidized to sulphate by mitochondria by the action of sulphite oxidase (SOX; also referred to as SUOX), in a mechanism that can contribute electrons to the electron transport chain via cytochrome c 34, 35. A less established route for hydrogen sulphide catabolism may involve reaction with iron‐heme species present, for example, in red blood cells and the mitochondrial cytochrome c oxidase 36, 37. In vitro, the products of heme‐mediated oxidation are polysulphides and thiosulphate, rather than sulphite 38. It is noteworthy that sulphate, sulphite, thiosulphate and polysulphide species all have significant bioactive properties, extending the potential physiological impact of cysteine metabolism and hydrogen sulphide production to the activities of these metabolites 39, 40, 41, 42.

A number of distinct mechanisms of hydrogen sulphide action have been proposed for regulating cellular function. One mechanism of action is regulation of the anti‐oxidant status of cells. The low micromolar concentrations of endogenous hydrogen sulphide, compared to the millimolar concentrations of more established thiol antioxidants such as glutathione and cysteine (free and protein‐bound), suggest that hydrogen sulphide likely plays an indirect role in the antioxidant capacity of cells through its signalling functions 43. In an early study of this property, primary cortical neurons were protected from glutamate‐induced cell death by administration of 100 μM sodium hydrogen sulphide (NaHS), an inorganic source of hydrogen sulphide 44. In that study hydrogen sulphide increased cystine import and glutathione production. There is also emerging evidence that hydrogen sulphide interacts with nitric oxide signalling 45, 46. Hydrogen sulphide reacts with nitric oxide to form nitrosothiol, and in some circumstances may stimulate cGMP signalling as may nitric oxide. Endogenous production of nitrosothiol was also measured in that study, suggesting a physiological role for direct sulphide–nitric oxide reactions. Modification of proteins by sulphydration (or persulphidation) is a further mechanism by which hydrogen sulphide may alter protein function. This modification refers to the presence of an additional sulphur atom attached to a thiol, such as cysteine, essentially a persulphide group. Formation of persulphides through hydrogen sulphide requires either oxidation of the hydrogen sulphide prior to reaction with a thiol, or reaction of hydrogen sulphide with an already oxidized species, such as a disulphide group 47, 48, 49. Sulphydration modification regulates the activity of ATP sensitive potassium channels, other membrane ion channels, kinases and transcription factors 48, 49 to elicit diverse biological effects. A persulphide cysteine is a known intermediate of the mercaptopyruvate sulphurtransferase enzyme catalytic cycle 50, suggesting that persulphide formation may increase reactivity of other active site cysteines.

## Hydrogen sulphide in the liver: enhancing glucose production and lipid oxidation?

Investigation into whether, and how, sulphide influences hepatic function is at an early stage. Existing data support the view that sulphide promotes glucose production, enhances lipid oxidation and inhibits insulin action 51, 52.

In the study by Zhang *et al* 51, treatment of clonal HepG2 hepatocyte cells with 10–100 μM NaHS reduced glucose consumption and reduced glycogen levels in association with reduced glucokinase activity. In contrast, phosphoenol pyruvate carboxykinase (PEPCK) activity and gluconeogenesis rates were increased by similar concentrations of NaHS in these cells. Notably, NaHS blocked stimulation of glucose consumption following insulin treatment, and this was associated with a reduction in phosphorylation (activation) of the insulin‐signalling intermediate AKT. A feedback mechanism between hydrogen sulphide and insulin signalling was uncovered, with insulin exposure reducing CSE levels and hydrogen sulphide production. Reversal of this regulatory loop was observed when the HepG2 cells were rendered insulin‐resistant through chronic culture in high glucose. In these cells, CSE protein was increased, as was hydrogen sulphide production.

*In vivo*, Wu *et al* 52 reported that daily injections of 50 μmoles/kg NaHS administration during a 16 week high‐fat diet regime prevented diet‐induced liver damage and increased markers of lipid oxidation. Specifically, hepatic expression of fatty acid synthase was reduced and expression of carnitine palmitoyltransferase‐1 was increased. High‐fat diet‐induced oxidative damage, as assessed by measuring hepatic malondialdehyde, was reduced in the NaHS‐injected mice, consistent with the findings of increased superoxide dismutase and glutathione peroxidase activity.

Taken together, hydrogen sulphide appears to oppose insulin action in the liver, and may mechanistically contribute to hepatic insulin resistance. Intriguingly, by enhancing glucose production and lipid oxidation, hydrogen sulphide mimics the actions of glucagon and/or adrenaline. This property fits with the increased production of hydrogen sulphide found in mice under dietary restriction 53 and clinical findings that hydrogen sulphide levels are low in obesity and type 2 diabetes 20, 21. This is consistent with the loss of potentially beneficial lipid oxidation effects and dysregulated hepatic lipid metabolism in these states. However, from the mechanistic studies above, low hydrogen sulphide levels would predict improved insulin sensitivity with respect to hepatic glucose homeostasis, which is clearly not the case in human type 2 diabetes. A full understanding of the potentially distinct regulation of glucose and lipid homeostasis by hydrogen sulphide signalling will be important to elucidate. A clearer understanding of the relationship between enzyme levels for hydrogen sulphide production, breakdown and the consequent steady‐state levels in liver and other key metabolic tissues in diabetes and obesity is also of critical importance.

## Hydrogen sulphide in adipose tissue: enhancing glucose disposal and increasing conditions for lipid storage?

Early studies with primary rat adipocytes suggested that hydrogen sulphide prepared as a saturated gas solution, and used effectively at doses in the range 10–1000 μM, could inhibit glucose uptake, thereby opposing insulin action 55. This finding has not been consistently replicated 56, 57, albeit some of the work in these later studies used NaHS, or sodium sulphide (Na_2_S) as a means of introducing hydrogen sulphide to their systems.

Studies of differentiated 3T3‐L1 adipocytes have generally supported the pro‐insulin and pro‐fat storage effects of hydrogen sulphide. High‐glucose culture conditions of differentiating 3 T3‐L1s, associated with insulin resistance, was found to reduce protein levels of molecular markers of the insulin‐signalling pathway, including PI3Kinase and phospho‐AKT as well as PIP3 levels 56. These effects were reversed by 10–100 μM Na_2_S, 100–1000 μM cysteine or the addition of insulin to the medium. The same authors also showed that reducing the insulin receptor level using siRNA blocked these effects of Na_2_S on signal transduction. In another study, high‐glucose medium conditions were found to reduce *CSE* mRNA, coincident with reduced production of the insulin sensitizer adiponectin and increased production of the pro‐inflammatory factor MCP‐1 57. The addition of 10–50 µm NaHS or forced expression of CSE reversed these effects. Finally, treatment with the compound GYY4137 (50 μM) or NaHS (50 μM) throughout the differentiation of 3 T3‐L1 cells led to the increased formation of lipid droplets and inhibition of adrenergic receptor‐stimulated lipolysis 58. Consistent with these findings, administration of GYY4137 at 200 μmoles/kg/day to mice reduced lipolysis, although no change in adiposity was observed 59. GYY4137, formally (*p*‐methoxyphenyl)morpholino‐phosphinodithioic acid, is claimed to release hydrogen sulphide slowly, and is used in an effort to mimic longer‐0term, and potentially more physiological, levels of the gas. However, the backbone structure of a donor such as GYY4137 could contribute to cellular effects, and how this is controlled for is not clear in this and similar studies. There are concerns over how to manipulate hydrogen sulphide levels in a physiologically appropriate manner, and the emergence of new donors will help future studies in this regard 60.

Overall, a model whereby hydrogen sulphide promotes insulin effects in the adipocyte could be proposed through its anti‐lipolytic action, although with respect to glucose uptake and lipogenesis its role is less clear. Hydrogen sulphide may also contribute to fat storage by indirectly maintaining or supporting insulin sensitivity in this tissue. Cysteine, when it has been studied in the context of adipose function, largely mimics the effects of hydrogen sulphide 54, 55, 58 and supports the hypothesis that it is a pro‐obesogenic factor. The notion that hydrogen sulphide may mediate some of these effects is perhaps not itself surprising; however, this does not easily fit with the initial findings that obesity is correlated with low plasma levels of hydrogen sulphide.

## Cysteine, hydrogen sulphide and insulin‐secreting pancreatic β cells

Over the last decade, hydrogen sulphide and/or cysteine have been shown to regulate insulin secretion from pancreatic islets, or clonal insulin‐secreting cells such as MIN6, INS‐1E and HIT‐T15. A consistent finding is that either cysteine or hydrogen sulphide can inhibit insulin release from β cells, particularly following stimulation with glucose 61, 62, 63, 64, 65, 66. It has been proposed that cysteine may mediate its effects, in part, via production of hydrogen sulphide, as CSE and CBS are expressed in the whole pancreas 67. Indeed CSE is specifically induced in β cells by high glucose 65. Hydrogen sulphide, added as gas‐saturated solution to 100 μm, enhances K‐ATP channel opening, whereas NaHS at 100 μM inhibits L‐type VDCC calcium channels, both mechanistically consistent with reduction of insulin release 61, 68. Sulphydration of K‐ATP channels has been demonstrated 69 and may contribute to the effect of hydrogen sulphide on insulin secretion. Regulation of mitochondrial respiratory function *per se* by hydrogen sulphide may be of further importance, given the key role of the ADP:ATP ratio on the secretory function of islet cells. Apart from a role in regulating insulin secretion *per se*, hydrogen sulphide may regulate other aspects of β cell physiology, including viability. In the stretptozotocin‐induced diabetes model, elevated hydrogen sulphide was implicated in contributing to β cell failure 70 and hydrogen sulphide prepared as saturated gas solution, administered at doses of 50–200 μM, were found to decrease the viability of INS‐1E cells in culture 71. In contrast, NaHS used at 100 µm was found to have a protective role in maintaining islet function and viability following high‐fat feeding in rats 72. Differences in the dose and duration of exposure may be a factor determining the overall effect of hydrogen sulphide on islet function, as in other tissues. Regulation of endogenous hydrogen sulphide production is also a complex process. Exposure to 20 mm glucose decreased hydrogen sulphide production in INS‐1E insulinoma cells 61, whereas similar glucose exposure stimulated expression of CSE in primary mouse islets 65. Plasma levels of hydrogen sulphide in a variety of diabetic models also reveals a complex picture. Lowered 73, 74, 75 and elevated 76, 77 levels have been reported in blood or plasma of type 1 and type 2 diabetes models. Even considering type 2 diabetes models exclusively, the aetiology of the diabetic state is different across the studies and the methodologies used to derive blood or plasma measurements of hydrogen sulphide are discordant, and thus interpretation remains somewhat controversial. If the current consensus, that hydrogen sulphide suppresses insulin secretion, holds, then the low hydrogen sulphide levels found in the plasma of type 2 diabetic patients 21, 22 may be a compensatory response. This may be permissive for the development of hyperinsulinaemia, which is required to maintain normal glucose levels in an insulin‐resistant state. It appears a critical issue to clarify the effects of hydrogen sulphide on islet function, development and dysfunction in diabetes.

## Lessons from genetic models with altered cysteine and sulphide metabolism

Complementing pharmacological and association studies, genetic models relevant to the production and metabolism of cysteine have been informative. So far, there has been a tendency to find that in those models resulting in lowered plasma total cysteine levels, lower adiposity is also observed whereas lean mass is largely unchanged 78. Notably, few of these models have been studied with respect to their hydrogen sulphide levels. The models summarized below provide insight into the regulation of, and phenotypic consequences of, cysteine and hydrogen sulphide perturbation.

### The transulphuration pathway: CBS and CSE

Insights into the metabolism and physiology of cysteine have been obtained from the study of transgenic manipulation of the genes *Cbs* and *Cse*. Mice homozygous for a knockout of the *Cbs* gene (*Cbs* ^−/−^) rarely survive after age 5 weeks 79. For this reason, most studies report on *Cbs* deficiency, rather than full knockout of the gene. By studying heterozygotes 80 or *Cbs* ^−/−^ that transgenically express a hypermorphic human CBS enzyme 81, *Cbs* ^−/−^ have been studied but, by necessity, experiments are restricted to very young mice 82. *Cse* gene knockout (*Cse* ^−/−^) is not associated with gross abnormality or lethality unless cysteine is limited in the diet 82, 83. Genetic backgrounds can significantly influence the phenotypic observations made with these genetic models. Lethality before adulthood of the *Cbs* ^−/−^ is observed on a C57BL/6 J, DBA/2 J or BALB/cA background, whereas on a C3H/HeJ background significant numbers of pups survive 84. Indeed, *Cbs* ^−/−^ mice that survive on the C3H/HeJ genetic background lose many of the phenotypic aspects considered typical for this model, suggesting induction of a compensatory system.

In spite of these caveats, some consistent findings relating to cysteine metabolism are summarized here. Knockout or deficiency of either *Cbs* or *Cse* in mice alters cysteine metabolism, consistent with their role in cysteine production from methionine. In some studies, this is reflected by lowered plasma or tissue levels of cysteine in *Cbs* [85a] and *Cse*‐knockout or ‐deficient mice 83, 86. Hyperhomocysteinaemia is another consistent finding in these genetic models 80, 82, 87. Notably, homocysteine is a long‐established risk factor for vascular disease when elevated 88. Another prediction of lowered cysteine production in tissue is reduced taurine. Cysteine dioxygenase (CDO) activity in liver, a first step in taurine synthesis, is increased when cysteine is elevated 89. Consistent with this, *Cse* ^−/−^ and *Cbs* ^−/−^ mice have low plasma taurine compared to control mice 82. Also consistent with a deficiency in cysteine, glutathione levels are lower in the liver of *Cbs* ^−/−^ 85 and *Cse* ^−/−^ 83 mice. Moreover, in the *Cse* ^−/−^ model, glutathione levels were normalized when cysteine was supplemented in the diet 83.

An expectation from *Cbs‐* and *Cse*‐deficient models is reduced hydrogen sulphide synthesis. In some studies this is the case. Lower plasma hydrogen sulphide levels have been reported in *Cse* ^−/−^ 83, 86, 87 and *Cbs* heterozygote mouse models 80. The expected reduction in hydrogen sulphide levels is not always observed in these models, for example liver hydrogen sulphide levels were no different from controls in *Cse* ^−/−^ 87. Furthermore, in that study protein levels of MPST, capable of hydrogen sulphide production, were increased. Moreover, sulphide quinone oxidoreductase (SQR) levels, important for oxidation of hydrogen sulphide, were elevated in this *Cse* ^−/−^ model, not expected in a model of hydrogen sulphide insufficiency. These findings highlight that a complex interplay and feedback mechanism(s) exists to regulate hydrogen sulphide signalling and the resultant phenotypic consequences.

Of relevance to adiposity, in one study of *Cbs* ^−/−^ hypomorphic mice, weight gain and in particular fat mass were lower than controls 90. When maintained on a high‐cysteine diet, *Cse* ^−/−^ mice are typically of normal body weight. On a cysteine‐restricted diet, however, weight loss is apparent 82, 83, including reduced fat mass 83. Supplementing drinking water with cysteine (1 mg/ml) partly reversed this weight loss, whereas daily intraperitoneal injection of NaHS (39 µg/kg), did not 83. A more recent study of *Cse* ^−/−^ mice on an atherogenic diet also found reduced weight gain compared to controls 91.

Little is known about glycaemic control in the *Cbs* and *Cse* knockout models. Very young (2 week‐old) *Cbs* ^−/−^ mice have lower plasma glucose levels, whereas comparably young *Cse* ^−/−^ mice do not 92, a finding that may simply reflect the severity of the *Cbs* ^−/−^ phenotype and makes assessment of the direct effects of hydrogen sulphide on glucose homeostasis difficult. Hepatic glycogen was higher in fed or mildly fasted *Cse* ^−/−^ mice, implying enhanced glucose storage in this tissue 51. Indeed, primary hepatocytes from these *Cse* ^−/−^ mice exhibited increased glucose uptake and reduced gluconeogenesis when compared to control hepatocytes. Moreover, islets from *Cse* ^−/−^ mice secreted more insulin in response to glucose 68. In contrast to these metabolically favourable phenotypes, another group reported that *Cse* ^−/−^ mice fed a high‐fat diet developed more severe glucose intolerance after 6 months 72. Given the complexity of changes attributable to lack of CSE enzyme activity, it remains unclear whether a change in hydrogen sulphide production is a primary mediator of altered glucose homeostasis.

More established, perhaps, are the profound effects on lipid metabolism and inflammatory pathology found in the liver of *Cbs* and *Cse* genetic models. Livers of *Cbs* ^−/−^ hypomorphic mice develop hepatic fibrosis and steatosis 79, 93. In another study, *Cbs* ^−/−^ hypomorphic mice showed increased hepatic mRNA levels for genes encoding lipid synthesis enzymes, and genes involved in the endoplasmic stress response 94. *Cbs* ^−/−^ hypomorphic and *Cbs* ^−/−^ mice are reported to have lower plasma levels of HDL and higher LDL, non‐esterified fatty acids and triglycerides 82, 95. A reduction in β‐oxidation of fatty acids is part of the mechanism for these phenotypes 95. By contrast, *Cse* ^−/−^ mice are protected from oxidative damage in the liver following lipopolysaccharide and galactosamine challenge 87. *Cse* ^−/−^ mice in some models appear normal with respect to lipid phenotypes 82. However, *Cse* ^−/−^ mice exposed to an atherogenic diet had higher total cholesterol levels, lower plasma triglycerides 91 and developed more extensive atherosclerotic lesions in their vessels. These phenotypes were reversed, in part, by intraperitoneal injection of NaHS (39 µg/kg), supporting that attenuated production of hydrogen sulphide in *Cse* ^−/−^ mice is an important factor in disease development. Of note, the correction of lipid abnormalities in this study did not prevent development of lesions in *Cse* ^−/−^ mice, further implicating vessel‐specific hydrogen sulphide signalling in atherosclerosis.

It is clear that CBS and CSE have roles relevant to glucose and lipid metabolism in the liver, and perhaps throughout the body, and have the potential to influence adiposity and diabetes. The relationship of reduced adiposity found in the gene deficiency models fits the clinical relationship with plasma cysteine, but less so with that reported for plasma hydrogen sulphide. Further work is required to clarify these important relationships.

### Reductive sulphide production: MPST

Another established source of endogenous sulphide is from the reductive removal of persulphide at the active site of 3‐mercaptopyruvate sulphur transferase (MPST) 96. 3‐Mercaptopyruvate (3‐MP) is a product of the transamination of cysteine. MPST then acquires the sulphur from 3‐MP as a persulphide into its active site, which, in combination with reduced thioredoxin, can be released as hydrogen sulphide. A *Mpst* ^−/−^ mouse has been generated, but no reports exist from this model describing cysteine, sulphide or phenotypes relating to adiposity, carbohydrate or lipid metabolism 97.

### Methionine cycle: PEMT and BHMT

The methionine cycle, upstream of the transulphuration pathway, uses methionine as a source of methyl groups for various reactions. This includes synthesis of phosphatidyl choline, which is catalysed by phosphatidylethanolamine *N*‐methyl transferase (PEMT). Subsequent to this, the cycle generates homocysteine, from which cystathionine, and subsequently cysteine, can be formed. Homocysteine can also be methylated to regenerate methionine. One enzyme capable of this methylation is betaine homocysteine methyltransferase (BHMT). Knockout mice for both *Pemt* and *Bhmt* have been generated. *Pemt* ^−/−^ mice show a protected metabolic phenotype, resisting the weight gain and deterioration of glucose metabolism caused by a high‐fat diet 98, 99. Apart from having lower homocysteine levels in plasma 100, no data exist regarding tissue or plasma cysteine in *Pemt* ^−/−^ mice. The *Bhmt* ^−/−^ mouse exhibits lower plasma cysteine, reduced fat mass, smaller adipocytes and improved glucose disposal following insulin injection 101. The involvement of hydrogen sulphide in these phenotypes remains to be investigated. However, methionine cycle intermediates are known to activate CBS activity 102, consistent with a potential impact of the methionine cycle on sulphide generation and its effects.

### Glutathione turnover: GGT and GCLM

Glutathione offers a major source of cysteine in the cell, with steady‐state concentrations generally far greater than that of cysteine. The production of glutathione from cysteine is contributed to by glutamate–cysteine ligase (GCL). A subunit of this complex (GCLM) has been knocked out in mice and results, as expected, in lower plasma and tissue glutathione but also in greatly reduced plasma cysteine 103. Strikingly, these *Gclm* ^−/−^ mice appear significantly affected in their adipose and liver metabolism; displaying a higher metabolic rate despite impaired whole‐body lipid oxidation, reduced fat storage in adipose, reduced fat accumulation in liver, greater oxidative stress, yet maintained insulin sensitivity on a high fat diet. Another study of this model found lower mRNA levels for genes involved in fatty acid oxidation and synthesis, as well as a profound protection from the development of hepatic steatosis 104.

Another enzyme relevant to glutathione turnover is γ‐glutamyl transferase (GGT), which is involved in recycling glutathione to cysteine. The enzyme participates in a number of other reactions and cannot be considered uniquely related to cysteine metabolism. *Ggt^−/−^* mice display alterations in glutathione and lower cysteine in plasma, suggesting that recycling of cysteine from glutathione represents an important source of the amino acid in tissues. Fat mass changes were not reported, but the mice were significantly reduced in body weight 105.

### Taurine biosynthesis: CDO and CSAD

Taurine is an essential and abundant metabolite of cysteine. The first step in its synthesis is by oxidation of a cysteine with molecular oxygen by the enzyme cysteine dioxygenase. The phenotype of mice with knockout of the cysteine dioxygenase gene (CDO) does not follow the predictions relating to cysteine levels and adiposity. Thus, while *Cdo* ^−/−^ mice have elevated cysteine levels, as expected, the mice exhibit lowered fat mass 106. However, hydrogen sulphide levels are elevated in *Cdo* ^−/−^ mice, which does fit the reported clinical relationship between hydrogen sulphide and obesity. The phenotype of *Cdo* ^−/−^ mice may be due to the toxicity of hydrogen sulphide and other altered metabolites, rather than a metabolic influence on fat mass *per se* 107. Cysteine sulphinic acid decarboxylase (CSAD) follows on from CDO in the taurine biosynthesis pathway, converting cysteine sulphinate to hypotaurine. CSAD may prevent accumulation of sulphite – a spontaneous breakdown product of cysteine sulphinate. A knockout mouse has been generated 108 but the result is lethal unless neonates are supplemented with taurine, and few data exist regarding any effects on metabolism in general.

## Genetic modification of the sulphide oxidation unit (SOU) represents a target for manipulating sulphide levels

While the genetic models discussed above largely concern cysteine metabolism or cytoplasmic sulphide generation, mitochondria are known to oxidize hydrogen sulphide, in part contributing to respiration, but also to prevent toxic build‐up. The known components of this system have been referred to as the sulphide‐oxidizing unit (SOU). The SOU consists of the sulphide quinone reductase (SQRDL), sulphur dioxygenase (SDO/ETHE1) and thiosulphate sulphur transferase (TST/rhodanese) 32, 33. In addition, sulphite is oxidized further to sulphate through sulphite oxidase (SUOX) 34. Together, this system is capable of oxidizing sulphide into sulphate and thiosulphate 109. As the system is predicted to lower hydrogen sulphide levels by oxidation via respiration, it is not surprising that the *Ethe1^−/−^* mouse, a model of ethylmalonic encephalopathy, is characterized by hydrogen sulphide levels in liver and muscle > 10‐fold higher than in control mice, and by a short life span 110. As yet, no reports exist for genetic manipulation of either the *SQRDL* or *SUOX* genes, although they have been studied in other ways and are suggested to be essential for the detoxification of sulphide and sulphite, respectively 111, 112. New studies on mice with gene deficiency of *Tst* are emerging, with phenotypes in the context of ischaemia–reperfusion injury in the heart (outlined in Abstract form 113). A full analysis of phenotypes related to metabolic disease are currently ongoing in our laboratory and will add to the increasingly recognized role for enzymes in the hydrogen sulphide breakdown pathway in many physiological processes.

## Summary of findings in genetic models of altered sulphur metabolism with respect to obesity

Genetic and biochemical investigations have delineated many of the important enzymes involved in the metabolism of cysteine and hydrogen sulphide (Figure 2). In the context of obesity, a number of the gene knockout models presented above mirror the clinical findings that obesity is associated with high plasma total cysteine. Gene knockout of enzymes of the methionine cycle (in particular *Bhmt*), the transulphuration pathway (*Cbs* and *Cse*) and glutathione metabolism (*Gclm* and *Ggt*) all reduce body weight, usually along with reduced adiposity, and present with lowered plasma cysteine. As yet, no genetic model has been described linking elevated cysteine to higher adiposity. Indeed, the *Cdo* knockout mouse has been shown to have significantly higher plasma cysteine and yet reduced body weight.

The clinical finding that plasma hydrogen sulphide correlates negatively with obesity is less well supported by the evidence from transgenic models. Of the models discussed, the *Cbs*, *Cse*, *Cdo* and *Sdo* genetic models have, in some studies at least, reported changes in plasma or tissue hydrogen sulphide, in the direction that would be predicted by their biochemical functions. However, in models where lower plasma hydrogen sulphide are found, such as the *Cse‐* and *Cbs‐*deficient or ‐knockout mice, lower rather than higher fat mass has been observed. The *Cdo‐* and *Sdo*‐knockout mice, on the face of it, support the clinical relationship, as they have elevations in hydrogen sulphide and lower body weight. These elevations in hydrogen sulphide are, however, reported in tissue, not plasma, and little has been reported about fat mass *per se*. Regardless of the similarity or otherwise to the clinical findings, caution must be exercised in translating adipose and whole body weight phenotypes of genetic models. Phenotypes that result from toxicity, as has been suggested for both the *Sdo* and *Cdo* knockout, and some *Cbs* and *Cse* models, greatly hamper interpretation.

The majority of models discussed above have a major impact on sulphur amino acid metabolism, including of cysteine and homocysteine. Genetic manipulation of components of the SOU may allow assessment of the contribution of hydrogen sulphide, and potentially sulphite, thiosulphate and sulphate, to lipid and carbohydrate metabolism, without necessarily interfering with cysteine metabolism directly.

## Summary of findings in genetic models of altered sulphur metabolism with respect to diabetes

Some of the above findings, in agreement with the existing clinical data, suggest that lowered cysteine is associated with impaired glucose homeostasis or insulin resistance. Few of the studies of the gene knockout models discussed above test this concept directly. Perhaps significantly, *Bhmt* ^−/−^ mice show lower cysteine levels and reduced adiposity, but improved glucose tolerance and insulin sensitivity. This association between lower plasma cysteine does not support the clinical data. Knockout of the transulphuration enzymes CSE and CBS present with pathology in the liver that might be considered diabetogenic; however, strict assessment of glucose homeostasis and insulin sensitivity is still lacking. Similarly, GCLM knockout mice, while presenting with lower cysteine, glutathione and increased oxidative stress, are reported to be comparable to controls regarding insulin sensitivity. With respect to hydrogen sulphide level, as data are lacking from the some of the above models, little can be said regarding its role in the observed phenotypes relating to diabetes or insulin function. In those knockout models where hydrogen sulphide levels are reported (*Cbs*, *Cse*, *Cdo* and *Ethe1*), a full investigation of potential diabetic phenotypes has not been reported. There therefore remains considerable uncertainty regarding the genetics of cysteine/sulphide‐metabolizing enzymes with respect to insulin resistance or diabetes.

## Conclusions and future directions

The association between obesity and cysteine in humans appears robust. However, it remains possible that the true mechanism underlying the association is a factor(s) other than cysteine, which concurrently drives elevations in plasma cysteine. Data on the dietary effects of cysteine and fat gain across a wide range of models is lacking. Differences between rodent strains, dietary sources and manipulation, the impact on microbiota and interactions with the non‐sulphur components of the diet may all influence how dietary cysteine effects adiposity. Similar issues apply to hydrogen sulphide's effects, which are obscured by a lack of standardization in measurement and administration methods. The field would benefit from a systematic analysis of tissue and blood/plasma sulphur metabolites across the key transgenic models. Robust methods for the measurement of hydrogen sulphide will be required in order to reflect true endogenous levels. The existence in blood or tissue samples of polysulphides 83, acid‐labile sulphur pools 114 and possible chemical interference by other factors can confound measurement techniques and result in wide variation in estimates of hydrogen sulphide 115. Tissue‐specific or inducible manipulation may be particularly informative to minimize toxicity and lethality that might result from whole‐body gene knockouts.

A full understanding of the impact of variation within the homologous human genes of the cysteine–sulphur metabolism pathway on metabolic parameters would also be informative. Nevertheless, exogenous administration of cysteine or hydrogen sulphide inhibits adrenergic‐stimulated lipolysis from adipocytes 14, 59, 60 and glucose‐stimulated insulin release from β cells 61, 62, 63, 64, 65, 66. Cysteine has further important effects on glutathione and taurine production in the liver, and thereby oxidative stress, lipid metabolism and inflammation. Regulation of these hepatic processes by hydrogen sulphide remains less clear, in large part due to the aforementioned lack of standardized methodology and variation in models and study designs. Direct dose‐sensitive effects of cysteine and sulphide on oxidative stress, mitochondrial respiration and metabolic rate *in vivo* will be important areas to resolve; the stimulatory effects of sulphide on mitochondrial respiration found with moderate, sub‐toxic doses remains an intriguing function of this gas, linked to protection of the mitochondria from ischaemia–reperfusion injury 116 and the 'cryoprotective‐like' properties first ascribed to hydrogen sulphide 117. Thus, although considerable work is needed to understand exactly how cysteine/sulphur metabolism relates to the development and potential treatment of obesity and diabetes, clear principles have been established, predominantly through work explored in the field of inflammatory and vascular biology, that these factors could be important and novel therapeutic targets for cardiometabolic diseases.

## Author contributions

RC wrote the manuscript and NMM edited it.
